# Early carbohydrate antigen 125 as a mortality predictor in hospitalized patients with coronavirus disease 2019

**DOI:** 10.3389/fcvm.2022.941512

**Published:** 2022-10-20

**Authors:** Oscar Moreno-Perez, Julio Nuñez, Miriam Sandin-Rollan, Vicente Arrarte, Vicente Boix, Sergio Reus, Hector Pinargote-Celorio, Isabel Ribes, Rocio Alfayate, Maria Belen Llorca-Santos, Maria Angeles Martinez-Garcia, Pablo Chico-Sánchez, Esperanza Merino

**Affiliations:** ^1^Department of Endocrinology and Nutrition, Alicante General University Hospital - Alicante Institute for Health and Biomedical Research (ISABIAL), Alicante, Spain; ^2^Department of Clinical Medicine, Miguel Hernández University of Elche, Elche, Spain; ^3^Department of Cardiology Valencia Clinic University Hospital – INCLIVA, Valencia, Spain; ^4^Department of Medicine, Valencia University, Valencia, Spain; ^5^CIBER Cardiovascular, Instituto de Salud Carlos III, Madrid, Spain; ^6^Department of Cardiology, Alicante General University Hospital - Alicante Institute of Health and Biomedical Research (ISABIAL), Alicante, Spain; ^7^Unit of Infectious Diseases, Alicante General University Hospital - Alicante Institute of Health and Biomedical Research (ISABIAL), Alicante, Spain; ^8^Department of Internal Medicine, Alicante General University Hospital - Alicante Institute for Health and Biomedical Research (ISABIAL), Alicante, Spain; ^9^Department of Clinical Analysis, Alicante General University Hospital - Alicante Institute of Health and Biomedical Research (ISABIAL), Alicante, Spain; ^10^Department of Pneumology, Alicante General University Hospital - Alicante Institute of Health and Biomedical Research (ISABIAL), Alicante, Spain; ^11^Department of Preventive, Alicante General University Hospital - Alicante Institute for Health and Biomedical Research (ISABIAL), Alicante, Spain

**Keywords:** CA125, COVID-19, hospitalized, mortality, risk factors

## Abstract

**Background:**

Carbohydrate antigen 125 (CA125) is an indicator of inflammation, immune response, and impaired cardiac function. The aim was to investigate whether CA125 behaves as a biomarker of severity and poor clinical outcomes in hospitalized patients with coronavirus disease 2019 (COVID-19).

**Methods:**

Serum CA125 [Elecsys CA125 II assay-(Roche Diagnostics GmbH)] was measured in stored biobank samples from COVID-19 hospitalized patients between 01 March 2020 and 17 October 2021. Multiple logistic regression models were built to explore the association between CA125 and clinical outcomes [in-hospital all-cause mortality, need for invasive mechanical ventilation (IMV), or non-invasive respiratory support (non-IRS)], estimating odds ratios (ORs; 95% CI). The gradient of risk of CA125 was evaluated by fractional polynomials.

**Results:**

A total of 691 patients were included, median age of 63 years (50–76), men (57.2%), with high comorbidity. At admission, 85.8% had pneumonia. Median CA125 was 10.33 U/ml (7.48–15.50). The in-hospital mortality rate was 7.2%. After adjusting for confounding factors, CA125 ≥ 15.5 U/ml (75th percentile) showed an increased risk of death [OR 2.85(1.21–6.71)], as age ≥ 65 years, diabetes, and immunosuppression. Furthermore, CA125 as a continuous variable was positive and significantly associated with the risk of death after multivariate adjustment. The mean hospital stay of the patients with CA125 ≥ 15.5 U/ml was longer than the rest of the study population.

**Conclusion:**

CA125 in the first 72 h of hospital admission seems a useful biomarker of mortality in hospitalized patients with moderate–severe COVID-19. If our findings are confirmed, the wide availability of this biomarker would make easy its widespread implementation in clinical practice.

“Early CA125 measurement, widely available in routine clinical practice, seems a useful biomarker of disease severity and mortality risk in hospitalized patients with moderate – severe #COVID-19 #CVD @isabial_iis”

## Introduction

Coronavirus disease 2019 (COVID-19) can trigger an inflammatory process with a complex pathophysiology and affect the cardiovascular system directly or indirectly (1), with an impact on the course of the disease (2, 3). The mechanisms of cardiac injury are poorly understood (1). A disordered renin–angiotensin system (RAS) activity (4, 5), mediated by binding of SARS-CoV-2 to angiotensin-converting enzyme 2 (ACE2) receptors present in the pulmonary alveoli, vascular and myocardial endothelial cells, could cause a direct cytotoxic effect on these cells (1, 6), besides triggering a severe systemic inflammation and cytokine storm (3, 7) that leads to respiratory dysfunction, myocardial and microvascular lesions (1), and the exacerbation of preexisting heart disease (8).

Early diagnosis and timely intervention in critical cases are crucial, highlighting the unmet need for novel biomarkers to improve diagnostic accuracy, risk stratification, monitoring, and therapy guidance.

The increases in cardiac and inflammatory biomarkers in COVID-19 have been associated with poor prognosis and mortality (9). Carbohydrate antigen 125 (CA125) has emerged as a useful and widely available marker in patients with decompensated heart failure (HF) (10). HF condition is closely related to systemic inflammatory activity and congestion (hydrostatic pressure and serosal effusions). Congestion is a hemodynamic parameter and causes the disease to progress by integrating into the circle of the inflammatory process defined in HF (11). Activation of mesothelial cells in response to increased hydrostatic pressures, mechanical stress, and cytokine activation has been suggested to be the crucial mechanism being the synthesis of CA125 by mesothelial cells (12). Although CA125 biological role is not well understood, it appears to be involved in multiple pathways, including immune innate and adaptive responses (13, 14).

Given that CA125 is not a specific cardiac biomarker, coupled with the fact that systemic inflammation has emerged as an important factor in increased CA125 concentrations (15), it is biologically plausible that this biomarker is useful in diseases in which inflammation is an important mechanism in the pathogenesis. In this regard, a correlation between CA125 and certain proinflammatory cytokines, such as tumor necrosis factor (TNF)-α, interleukin (IL)-6, and IL-10, has been identified in HF (16). Also, in cultured human mesothelial cells, the secretion of CA125 can be enhanced by the inflammatory cytokine interleukin-1 beta (IL-1 beta), tumor necrosis factor-alpha (TNF-alpha), or lipopolysaccharide from *Escherichia coli* (17).

As a marker of inflammation, immune response, and cardiac function impairment, we postulated that CA125 may be useful for predicting unfavorable outcomes in patients with COVID-19. The availability of CA125 in most clinical laboratories, together with its standardized measurement and reduced cost, makes this marker attractive for routine use (18).

To provide insights into this issue, the impact of CA125 levels on major outcomes was examined in patients hospitalized with COVID-19.

## Methods

### Patients and study design

Since the beginning of the pandemic, every adult patient admitted to Hospital General Universitario Dr. Balmis de Alicante – a tertiary center – was asked for informed consent to be included in a database and to obtain a blood sample for biobank storage.

**FIGURE 1:**
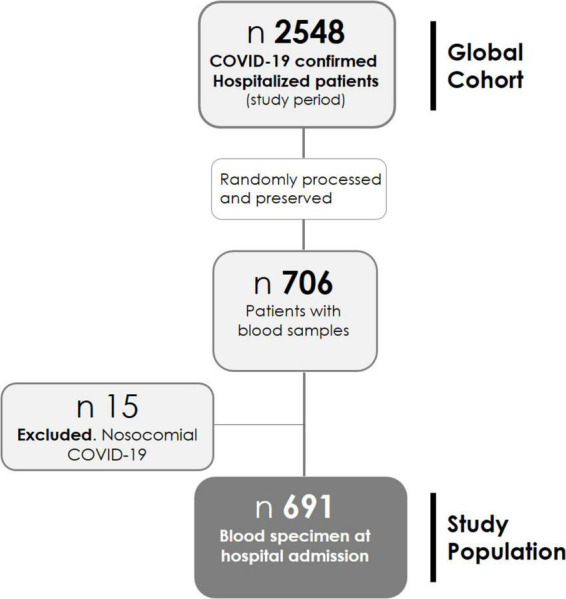
Flowchart of coronavirus disease 2019 (COVID-19) hospitalized in the study period.

Patients hospitalized between 01 March 2020 and 17 October 2021 are studied. Blood samples were collected in EDTA tubes; plasma was separated from whole blood by centrifugation at 3,000 × *g* for 15 min at 4^°^C, then aliquoted and frozen at −80^°^C until use, by the BioBank ISABIAL, and integrated with the Spanish National Biobank Network and with the Valencian Biobanking Network. From 2,548 patients admitted during the study period, samples from 706 patients were randomly processed and preserved (Figure 1). Those with nosocomial COVID-19 were excluded from this analysis (*n* = 15), leaving the study sample in 691 patients.

Inclusion criteria were as follows: age ≥ 18 years, not nosocomial confirmed SARS-CoV-2 infection by the RT-PCR-COBAS 6800 System (Roche Molecular Systems, Branchburg, NJ, USA), informed consent signature, and availability of biobank blood sample with extraction in the first 72 h after hospital admission.

### Variables and data collection

The clinical features, comorbidity, laboratory and radiological tests, prescribed therapies, and outcomes during the acute phase of the infection by SARS-CoV-2 were extracted from the digital medical records. The laboratory variables have been dichotomized, according to clinically relevant cutoff points or, failing that, according to the upper limit of the reference values of the center (9, 19–23). For the following variables, standard categorizations were followed: age ≥ 65 years, Charlson comorbidity index ≥ 3, estimated glomerular filtration rate < 60 ml/min/1.73 m^2^ (by CKD-EPI formula), and hypoxemia (oximetry < 94% and PaO2:FiO2 < 300 mmHg) (24). The Charlson index assigns weights for specific diseases and includes myocardial infarction, congestive HF, peripheral vascular disease, cerebrovascular disease, dementia, chronic pulmonary disease, ulcer disease, mild liver disease, diabetes with or without end-organ damage, any tumor, leukemia, lymphoma, moderate or severe liver disease, metastatic solid tumor, and AIDS.

### Measurements and definitions

Serum CA125 was measured from biobank samples, following standardized and reproducible methods of their processing, by electrochemiluminescence immunoassay [Elecsys CA125 II assay-(Roche Diagnostics GmbH, Sandhofer Strasse 116, D-68305 Mannheim)] and was dichotomized by its 75th percentile.

### Outcomes

The endpoints of this analysis were in-hospital all-cause mortality (main), need for invasive mechanical ventilation (IMV) or non-invasive respiratory support (secondaries), and associated factors.

### Statistical analysis

Categorical and continuous variables are given as frequencies (percentages) and as median (interquartile range), respectively. Mann–Whitney *U* and Chi-square tests were used for group comparisons. The correlation between explanatory variables was analyzed by Spearman’s Rho. Cumulative incidences of outcomes [95% confidence intervals (95% CI)] were registered.

Multiple logistic regression models were built to explore the association between CA125 and clinical outcomes, estimating odds ratios (ORs; 95% CI) in the global cohort and the subgroups. The variables were included as covariates if shown significant associations in simple models. The gradient of risk of CA125, as a continuous variable, in univariate and multivariate settings, was evaluated by fractional polynomials. The final covariates included in the multivariate model were as follows: ≥ 65 years of age, Charlson comorbidity > 3, sex, nursing home, confusion, diabetes, hypertension, immunosuppression, eGFR ≤ 60 ml/min/m^2^, oximetry at room air < 94%, ferritin > 500 mg/L, troponin T > 14 ng/L, B-type natriuretic peptide > 125 pg/ml, procalcitonin > 0.15 ng/ml, lactate dehydrogenase (LDH) > 250 U/L, C-reactive protein > 10 mg/dl, lymphopenia (< 1,000/mm^3^), and the exposure (CA125). The number of patients included in the multivariate analysis was 583 (84.4% of the initial sample). No multiple imputations were performed. Covariates with more than 15% missing were not included in the multivariate analysis. The discriminative ability of the models was assessed by ROC analysis. A specific model was built to study the association between CA125 and mortality in the oldest subpopulation (age ≥ 85 years).

All tests were two-tailed, and a *p*-value of less than 0.05 was used. IBM SPSS Statistics 25 and STATA 15.1 statistical packages were used for the analyses.

This project was performed in the Clinical and Biomedical Research Institute of Alicante (ISABIAL), under the written approval of the local Ethics Committee of Clinical Research (Reference 200379).

## Results

### Baseline characteristics

Of the 2,548 patients hospitalized in the study period, blood samples from 706 patients were available. Fifteen patients with nosocomial COVID-19 were excluded. Finally, 691 patients were included in this study (refer to the flowchart in Figure 1). The basal demographic characteristics, comorbidities, clinical presentation, and outcomes are shown in Table 1. For more detailed information, refer to Supplementary Table 1.

**TABLE 1:** Demographic characteristics, comorbidities, clinical presentation, and clinical outcomes.

	Total (*n* = 691)
**Demographics**	
Age (years), median (IQR)	62 (50–76)
Age > 65, %	320/691 (46.3)
Males, %	395/691 (57.2)
Vaccine status^a^ Complete Partial	35/691 (5.1%) 13/691 (1.9%)
**Comorbidities**	
Diabetes, %	155/691 (22.4)
Hypertension, %	328/691 (47.5)
Chronic respiratory disease	123/690 (17.8)
Smoker (current or former), %	53/538 (9.9)
Charlson comorbidity index ≥ 3, %	341/688 (49.6)
Obesity (BMI ≥ 30), %	190/479 (39.7)
**Initial assessment**	
Oximetry at room air < 94%, %	211/654 (32.3)
Lymphopenia (< 1000/mm^3^), %	332/691 (48.0)
Troponine T > 14 ng/L	216/643 (33.6)
Brain natriuretic peptide > 125 pg/ml, %	303/640 (47.3)
**Clinical presentation**	
Days of symptoms before admission, median (IQR)	6.8 (4–10)
Dyspnea, %	406/689 (58.9)
Radiological characteristics Bilateral pneumonia, % Unilateral pneumonia, %	236/683 (34.6) 350/683 (51.2)
Opacities > 50% of lung surface on X-Rays, %	155/691 (22.4)
**Clinical outcomes**	
Length hospital stay (days), median (IQR)	8 (5–12)
Non-invasive respiratory support, %	192/691 (27.8)
ICU admission, %	79/691 (11.4)
Length ICU stay (days), median (IQR)	17.2 (6–17)
Invasive mechanical ventilation, %	52/691 (7.5)
**Deaths, %**	
Global, %	50/691 (7.2)
Group with ≥ 85 years old, %	14/72 (19.4)
Group with < 85 years old, %	36/619 (5.8)
Group with IMV, %	15/52 (28.8)

ICU, intensive care unit; IMV, invasive mechanical ventilation; IQR, interquartile rate.

^a^We defined complete vaccination (CV) as symptom onset after 14 days of the second dose of vaccines (a single Janssen dose) and partial vaccination (PV) as administration of only the first dose, or symptom onset within 13 days after the second dose (single dose in Janssen).

The population was composed mainly of men (57.2%), with a median age of 63 years (50–76) and high comorbidity (Charlson index ≥ 3 46.6%, hypertension 47.5, obesity 39.7%, and diabetes 22.4%). Notably, 5% had received a complete vaccination (at least 14 days before the onset of clinical symptoms). After a mean of 1 week of symptoms, they were admitted to hospital, with hypoxemia in 32.3% and pneumonia in 85.8% of cases (bilateral pneumonia 34.6%; opacities > 50% of lung surface 22.4%). At admission, 33.6 and 47.3% of patients had T-Troponin > 14 ng/L and pro-BNP > 125 pg/ml, respectively.

Endotracheal intubation was required in 7.5% (52/691) of the patients. The in-hospital mortality rate was 7.2% (50/691). Biobank samples were obtained in 62.4 and 86.1%, in the first 24 and 48 h of hospital admission, respectively. Median CA125 was 10.33 U/ml (7.48–15.50).

### Carbohydrate antigen 125 and severity of the disease

Patients in the upper quartiles showed a worse baseline risk profile (Supplementary Table 2). They were older, more frequently women, and had more comorbidities, higher cardiac biomarker (troponin T and NT-proBNP) levels, and higher procalcitonin and ferritin, as shown in Supplementary Table 2.

There was a weak correlation between the levels of the natural logarithm (ln) CA125 with age (rho 0.21) amino terminal brain natriuretic peptide (NTpro-BNP) (rho 0.22) and T-troponin (rho 0.21), *p* < 0.001. At the same time, a correlation with IL-6 levels on admission was not observed.

### Carbohydrate antigen 125 and risk of death

Supplementary Table 3 shows the baseline characteristics across the death status. During hospitalization, 50 patients died (7.2%). Plasma CA125 was higher in patients with fatal outcome [14.35 U/ml (8.30–27.81) vs. 10.24 U/ml (7.44–14.98), *p* = 0.008]. The rates of in-hospital death were significantly higher in the upper CA125 quartile [Q1 (≤ 7.47 U/ml): 5.2%, Q2 (7.48–10.3 U/ml): 5.8%, Q3 (10.3–15.48): 4.1%, and Q4 (≥ 15.5 U/ml): 13.9%; *p* < 0.001].

**FIGURE 2:**
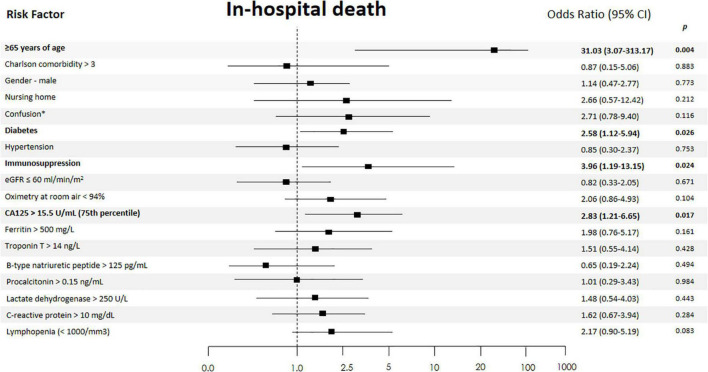
Independent risk factors of in-hospital death. The 95% confidence intervals (CIs) of the odds ratios have been adjusted for multiple testing. In bold, independent predictors associated with the outcomes. eGFR, estimated glomerular filtration rate (by CKD-EPI formula); *On admission.

**FIGURE 3:**
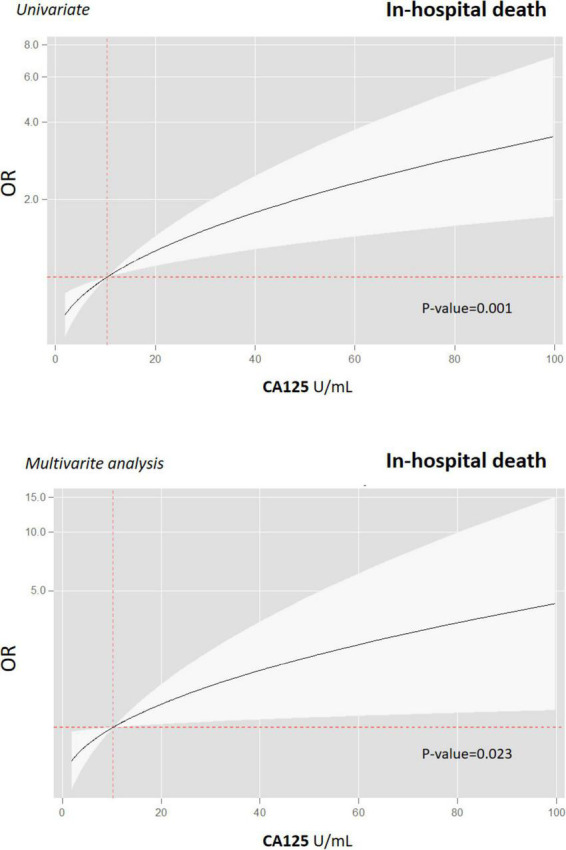
Gradient of risk of CA125 for predicting in-hospital death in patients with COVID-19. Prognostic effect of CA125 on in-hospital all-cause mortality and in univariate (upper graph) and multivariate analyses (lower graph). CA125, antigen carbohydrate 125; OR, odds ratios.

When CA125 was categorized in quartiles, those in the upper quartile showed a significantly increased risk [OR 2.94 (1.32–6.52)], compared with the lower quartile. In the multivariate regression model, after adjusting for confounding factors, when compared with the three lower quartiles (< 15.5 vs. ≥ 15.5 U/ml), those in the upper category remained to show an increased risk of death [OR 2.85 (1.21–6.71)], along with age ≥ 65 years [OR 30.4 (3.02–305.02)], diabetes [OR 2.54 (1.10–5.91)], and immunosuppression [OR 4.08 (1.20–13.85)] (Figure 2). Lymphopenia was close to statistical significance. Final multivariate risk estimates for all covariates included in the models are presented in Figure 2. After multivariate adjustment, CA125 as a continuous was positive and significantly associated with the risk of death (Figure 3).

Subgroup analysis revealed that those in the upper quartile vs. the three lower quartiles remained to show a homogenous increased risk of in-hospital death across age (< 65 vs. ≥ 65 years), sex (men vs. women), and Charlson index (< 2 vs. ≥ 3). The adjusted *p*-value for the interactions for those belonging to the upper quartile vs. three lower quartiles was 0.483, 0.189, and 0.586 for age, sex, and Charlson status, respectively. Table 2 shows the risk estimates for each subgroup.

**TABLE 2:** CA125 and adjusted risk of in-hospital death.

	OR (CI 95%)*	*p*-value for interaction
Upper quartiles vs. three lower quartiles (< 15.5 vs. ≥ 15.5 U/ml)		
**Whole sample**		
Whole sample	2.85 (1.21–6.71)	
**Age**		
< 65 years	2.21 (1.17–11.91)	0.483
≥ 65 years	3.12 (1.56–5.67)	
**Sex**		
Men	2.01 (0.95–4.18)	0.189
Women	4.35 (1.51–14.51)	
**Charlson index**		
< 2	1.95 (0.83–13.65)	0.586
≥ 3	3.21 (1.57–5.32)	

Subgroup analysis. *Adjusted estimates. CA125, antigen carbohydrate 125; OR, odds ratio.

After excluding patients aged ≥ 85 years, age ≥ 65 years, diabetes, and CA125 > 15.5 U/ml persist as independently associated factors of mortality, whereas confusion and hypoxemia at admission were close to statistical significance.

### Carbohydrate antigen 125 and other clinical outcomes

Carbohydrate antigen 125 levels were not associated with the need of IMV [CA125 > 50th percentile OR 0.87 (0.49–1.55), CA125 > 75th OR 0.72 (0.35–1.47)] or non-invasive respiratory support [CA125 > 50th percentile OR 0.88 (0.63–1.23), and CA125 > 75th OR 1.08 (0.73–1.28)].

The mean hospital stay of the patients with CA125 higher than the 75 h percentile was longer than the rest of the study population [9.0 (6.0–15.0) vs. 7.0 (5.0–11.0) days, *p* = 0.014].

## Discussion

This is the first study analyzing the role of CA125 in the first 72 h of admission as a biomarker of disease severity in hospitalized patients with moderate–severe COVID-19. CA125 was higher in patients with fatal outcomes, whereas did not entail a greater requirement of IMV. Even though pro-BNP, T-troponin, and CA125 correlated positively, these associations were weak. Our findings establish CA125 levels as a sensitive biomarker of severity and poor clinical evolution in hospitalized COVID-19.

Carbohydrate antigen 125, also called cancer antigen 125, carcinoma antigen 125, or mucin 16 (MUC16), is a complex glycoprotein encoded by the MUC16 gene in humans (13, 25). CA125 is mainly synthesized by mesothelial cells in the pericardium, pleura, or peritoneum (25, 26). In recent years, increasing evidence supported the use of CA125 in cardiovascular diseases, particularly in decompensated HF and in the transition to clinical stability (10, 27). Interestingly, in patients with acute HF, this glycoprotein provides additional prognostic information to those provided by well-known prognosticators, including natriuretic peptides (28).

### Pathophysiology of the association between CA125 and death in COVID-19

In different CV scenarios, especially in acute HF, plasma levels of CA125 have emerged as proxies of two crucial and interrelated pathophysiological processes, namely, inflammation and congestion (18). Thus, several works have found a positive and significant association between CA125 and surrogate parameters of fluid overload and right-sided HF dysfunction (29). Additionally, higher glycoprotein levels also identified patients with a greater immunoinflammatory milieu (18). For instance, Miñana et al. reported in a cohort of 132 patients admitted with acute HF that CA125 levels above the median (> 60 U/ml) were associated with higher levels of TNF-α, IL-6, and interleukin-1β and lower relative lymphocyte count (30). Also, Kosar et al. showed that the increase in serum CA 125 levels in 35 patients with HF correlates with TNF-α (*r* = 0.624, *p* < 0.001), IL-6 (*r* = 0.671, *p* < 0.001), and IL-10 (*r* = 0.545, *p* < 0.001) (16). These findings contrast with the lack of correlation between the levels of nlCA125 and IL-6 in our series. However, the patients in the upper quartiles of CA125 levels showed higher inflammatory markers such as procalcitonin and ferritin, reflecting the degree of underlying systemic inflammation. The short half-life of IL-6 (2–5 h) (31) and the analysis of only one sample at admission, in our study, and not sequential measurements could explain these discrepancies. In this regard, in patients without COVID-19 with systemic inflammatory response syndrome/sepsis admitted to ICU, Oda et al. demonstrated that there was no significant difference in the blood IL-6 level on admission between survivors and non-survivors, whereas the mean blood IL-6 level during ICU stay was significantly higher in the non-survivors (31).

We postulate that the mechanisms endorsing the relationship between CA125 and the risk of mortality in COVID-19 are due to at least two main pathophysiological mechanisms that partially overlap. First, we think that CA125 may capture the intensity of the inflammatory response more accurately than other inflammatory markers. Our findings positioned CA125 as an independent biomarker of fatal outcomes in patients hospitalized for COVID-19, above the classical inflammation markers described in the literature (ferritin, procalcitonin, LDH, and C-reactive protein) (32, 33) or biomarkers of myocardial damage (T troponin and pro-BNP) (9). CA125, in contrast to other biomarkers, is a stable biomarker that may capture the severity of immunoinflammatory response in a period of several days (the half-life of CA125 ranges from 5 to 12 days) (18). Second, CA125 may also capture information about the onset of clinical complications such as HF, pulmonary thromboembolism with right-sided dysfunction, or pleural effusion. This greater elevation in the most seriously ill patients could respond to a greater pulmonary involvement because CA125 concentrations are correlated with hemodynamic parameters, right atrial pressure, and pulmonary capillary wedge pressure (34).

### Limitations

Some important limitations need to be addressed. First, this is a one-center observational analysis of patients hospitalized with COVID-19. Although the effort to control for relevant confounders was performed, the risk of residual confounding, as a selection bias for available biobank samples, cannot be ruled out in this type of study. Sample size limitations prevented analysis by the SARS-CoV-2 variant. In this study, we only measured this glycoprotein at a one-time point. Thus, we could not explore the kinetic of this biomarker and its influence on risk stratification.

Several gaps are worth mentioning, the pathophysiology of CA125 upregulation in COVID-19 is not well known, and whether CA125 is a marker or plays a role in disease progression remains speculative. The optimal cutoff for defining severity should be corroborated in future research. Finally, we did not register a prior history of HF or cancer in the evaluated sample. CA125 is a well-established marker of different neoplasms. Therefore, we cannot assess its role as a confounding factor in the current findings.

### Future directions

These findings require to be validated in larger studies, and more research is needed to define its biological role in patients with COVID-19. The larger sample size may be helpful for confirming current findings and unraveling the clinical utility of the results presented in this study. Additionally, formal prognostic comparison among different inflammatory markers is still required. Further studies are warranted to determine the optimal set of widely available circulating biomarkers useful in patients with COVID-19. Whether CA125 will be among them remains to be confirmed. In the meantime, the usefulness of this biomarker in guiding the intensity of medical therapy seems a reasonable hypothesis that deserves further evaluation.

### Logistic advantages

Carbohydrate antigen 125 has potential logistic advantages that deserve to be highlighted. First, the wide availability of CA125 in most clinical laboratories, its measurement following standardized and reproducible methods, and low cost (< 2.5 € per determination) make this marker attractive for routine use in decompensated HF and other diseases. Second, CA125 levels are not substantially modified by age, sex, body mass index, or renal dysfunction. Furthermore, in all of the prior subgroups, plasmatic CA125 retained its prognostic value (18, 35). All of the prior items are crucial aspects that yield us to speculate an easy transition of these findings to the actual clinical practice of patients hospitalized with COVID-19.

## Conclusion

Carbohydrate antigen 125 measured in the first 72 h of hospital admission seems a useful biomarker of disease severity in hospitalized patients with moderate-severe COVID-19. Besides, this sensitive biomarker, as a surrogate of congestion and inflammation, may reflect the progression of COVID-19 and is independently associated with in-hospital mortality after adjusting by confounders. If our findings are confirmed, the wide availability of this biomarker will make easy its widespread implementation in clinical practice.

Further research is required to understand better its biological role and its promising utility as a prognostic marker in COVID-19.

## Data availability statement

The raw data supporting the conclusions of this article will be made available by the authors, without undue reservation.

## Ethics statement

This project was performed in the Clinical and Biomedical Research Institute of Alicante (ISABIAL), under the written approval of the Local Ethics Committee of Clinical Research (Reference 200379). The patients/participants provided their written informed consent to participate in this study.

## Author contributions

OM-P, JN, MS-R, VA, and EM: writing – original draft. OM-P, JN, MS-R, VA, VB, SR, HP-C, IR, RA, ML-S, MM-G, and EM: writing – review and editing, and investigation. OM-P, JN, and EM: methodology. OM-P and JN: formal analysis. EM: project administration. All authors contributed to the article and approved the submitted version.
