# Supplementary material for: Early carbohydrate antigen 125 as a mortality predictor in hospitalized patients with coronavirus disease 2019
Source: Front Cardiovasc Med. 2022 Oct 20;9:941512. doi: 10.3389/fcvm.2022.941512 (PMC9631479; doi:10.3389/fcvm.2022.941512)
Supplement: Supplementary file 1 [file Data_Sheet_1.docx]

**Suppl. Table 1. Complete basal demographic characteristics, comorbidities, clinical presentation, established therapies and outcomes.**

|  | Total  [n=691] |
| --- | --- |
| **Demographics** | |
| Age (years), median (IQR) | 62 (50-76) |
| Age > 65, % | 320/691 (46.3) |
| Males, % | 395/691 (57.2) |
| Long-term care resident, % | 15/691 (2.2) |
| Health professional, % | 26/691 (3.8) |
| Caucasian | 564/691 (81.6) |
| Central/South American | 82/691 (11.9) |
| Vaccine status^a^  Complete  Partial | 35/691 (5.1%)  13/691 (1.9%) |
| Pandemic Waves | |
| First (01.03.2020-30.04.2020)  Second (01.05.2020-31.08.2020)  Third (01.09.2020-30.11.2020)  Fourth (01.12.2020-31.05.2021)  Fifth (01.06.2021-17.10.2021) | 21.4%  2.2%  21.6%  43.1%  11.7% |
| Comorbidities | |
| Diabetes, % | 155/691 (22.4) |
| Hypertension, % | 328/691 (47.5) |
| Chronic respiratory disease | 123/690 (17.8) |
| Smoker (current or former), % | 53/538 (9.9) |
| Immunodepression, % | 51/691 (7.4) |
| Charlson comorbidity index, median (IQR) | 3.07 (1-5) |
| Charlson comorbidity index ≥3, % | 341/688 (49.6) |
| Obesity (BMI ≥30), % | 190/479 (39.7) |
| Initial assessment | |
| Oximetry at room air (%), median (IQR) | 94.2 (92-97) |
| Oximetry at room air < 94%, % | 211/654 (32.3) |
| PaO2:FiO2 ratio, median (IQR) | 372.4 (300-422.3) |
| PaO2:FiO2 ratio < 300, % | 380/498 (76.3) |
| Systolic BP (mmHg), median (IQR) | 133.2 (118-147) |
| Diastolic BP (mmHg), median (IQR) | 80 (70-90) |
| Heart rate (beats/min), median (IQR) | 93.3 (81-104) |
| Heart rate >100 beats/min, % | 235/676 (34.8) |
| Respiratory rate (breaths/min), median (IQR) | 20 (16-22) |
| Temperature (ºC), median (IQR) | 36.9 (36.2-37.6) |
| Lymphocytes (per mm^3^), median (IQR) | 1112 (755-1380) |
| Lymphopenia (<1000/mm^3^), % | 332/691 (48.0) |
| C-reactive protein (mg/dl), median (IQR) | 8.2 (2.7-11.5) |
| C-reactive protein > 10 mg/dl, % | 203/691 (29.4) |
| Procalcitonin (ng/mL), median (IQR) | 0.5 (0.06-0.17) |
| Procalcitonin > 0.5 ng/mL, % | 52/652 (8.0) |
| Ferritin (mg/L), median (IQR) | 945.8 (330-1289) |
| Ferritin > 500 mg/L, % | 406/673 (60.3) |
| Lactate dehydrogenase (U/L), median (IQR) | 310.5 (231.8-359) |
| Lactate dehydrogenase > 250 U/L, % | 438/654 (67.0) |
| D-dimers (mg/mL), median (IQR) | 3.37 (0.46-1.3) |
| D-dimers > 1 mg/mL, % | 219/656 (33.4) |
| Troponine T, ng/L | 22.7 (5-18) |
| Troponine T > 14 ng/L | 216/643 (33.6) |
| Brain natriuretic peptide (pg/ml), median (IQR) | 832.5 (37 -437.5) |
| Brain natriuretic peptide > 125 pg/ml, % | 303/640 (47.3) |
| Potassium (mmol/L), median (IQR) | 4.3 (3.9-4.5) |
| Potassium < 3.5 mmol/L, % | 62/542 (11.4) |
| Urea (mg/dl), median (IQR) | 46.4 (27-51) |
| eGFR (ml/min/m^2^), median (IQR) | 77.1 (61.6-90) |
| eGFR < 60 ml/min/m^2^, % | 161/689 (23.4) |
| IL6 (pg/ml), median (IQR) | 69.9 (9-63) |
| IL6 > 10 pg/ml, % | 455/621 (73.3) |
| Clinical presentation | |
| Days of symptoms before admission, median (IQR) | 6.8 (4-10) |
| Fever, % | 494/689 (71.7) |
| Cough, %  Dry cough, %  Wet cough, % | 208/688 (30.2)  401/688 (58.3)  104/688 (15.1) |
| Dyspnea, % | 406/689 (58.9) |
| Anosmia-dysgeusia, % | 131/677 (19.4) |
| Myalgias-arthralgias, % | 225/682 (33) |
| Fatigue, % | 324/680 (47.6) |
| Diarrhoea, % | 192/685 (28) |
| Confusion, % | 33/688 (4.8) |
| Radiological characteristics  Bilateral pneumonia, %  Unilateral pneumonia, % | 236/683 (34.6)  350/683 (51.2) |
| Opacities >50% of lung surface on X-Rays,% | 155/691 (22.4) |
| Treatment | |
| Remdesivir, % | 93/542 (17.2) |
| Dexametasone, % | 477/543 (87.8) |
| Another corticosteroid, % | 102/534 (19.1) |
| Tocilizumab, % | 225/688 (32.7) |
| Hiperimmune plasma, % | 11/691 (2.0) |
| Clinical Outcomes | |
| Length hospital stay (days), median (IQR) | 8 (5-12) |
| Non-invasive respiratory support, % | 192/691 (27.8) |
| Type of non-invasive respiratory support, %  HFNC, %  NIMV, % | 138 (71.9)  54 (28.1) |
| ICU admission, % | 79/691 (11.4) |
| Length ICU stay (days), median (IQR) | 17.2 (6-17) |
| Invasive mechanical ventilation, %  Global, %  Group with Age≥85 years old, %  Group with maximum care, % | 52/691 (7.5)  1/72 (1.4%)  51/619 (8.3) |
| Days of IMV, median (IQR) | 9 (6-25) |
| Readmitted, % | 28/691 (4.2) |
| Deaths, %  Global, %  Group with ≥85 years old, %  Group with <85 years old, %  Group with IMV, % | 50/691 (7.2)  14/72 (19.4)  36/619 (5.8)  15/52 (28.8) |

BMI: body mass index; BP: blood pressure; COPD: chronic obstructive pulmonary disease; CV: cardiovascular; eGFR: estimated glomerular filtration rate (by CKD-EPI formula); HFNC: high flow nasal cannula; ICU: intensive care unit; IL6: interleukin-6; IMV: invasive mechanical ventilation; IQR: interquartile rate; NIMV: non-invasive mechanical ventilation.

Laboratory reference upper limit of normal: C-reactive protein 0.5 mg/dL, D-dimers 0.5 mg/mL, Ferritin 150 mg/L, Lactate dehydrogenase 250 U/L, Troponin T 14 ng/L, CK 170 U/L, Procalcitonin 0.5 ng/mL, IL6 10 pg/ml, Pro-BNP categorized by age (< 50 years old, 450 pg/mL; 50–75 years old, 900 pg/mL; > 75 years old, 1800 pg/mL).

^a^ We defined complete vaccination (CV) as symptom onset after 14 days of the second dose of vaccines (a single Janssen dose) and partial vaccination (PV) as administration of only the first dose, or symptom onset within 13 days after the second dose (single dose in Janssen).

**Suppl. Table 2. Baseline risk profile and clinical outcomes by CA125 quartiles.**

| \|  \| 1 \| 2 \| 3 \| 4 \| Total \|  \| \| --- \| --- \| --- \| --- \| --- \| --- \| --- \| \|  \| (N = 172) \| (N = 173) \| (N = 173) \| (N = 173) \| (N = 691) \| p-value \| |
| --- | --- | --- | --- | --- | --- | --- | --- | --- | --- | --- | --- | --- | --- | --- |
| \|  \|  \|  \|  \|  \|  \|  \| \| --- \| --- \| --- \| --- \| --- \| --- \| --- \| \| **Demographics and Comorbidities** \| \| \| \| \| \| \| \| **Age (years),** \|  \|  \|  \|  \|  \|  \| \| Mean (SD) \| 59 (16) \| 60 (17) \| 61 (18) \| 68 (17) \| 62 (17) \| **<0.001** \| \| Median (Q1, Q3) \| 60.0 (46.0, 72.0) \| 61.0 (49.0, 74.0) \| 62.0 (50.0, 74.0) \| 69.0 (56.0, 82.0) \| 63.0 (50.0, 76.0) \|  \| \| N (% Missing) \| 172 (0.0%) \| 173 (0.0%) \| 173 (0.0%) \| 173 (0.0%) \| 691 (0.0%) \|  \| \| **Age ≥65 years old**  N (%) \|  \|  \|  \|  \|  \| **0.007** \| \| yes \| 70 (40.7%) \| 72 (41.6%) \| 79 (45.7%) \| 99 (57.2%) \| 320 (46.3%) \|  \| \| no \| 102 (59.3%) \| 101 (58.4%) \| 94 (54.3%) \| 74 (42.8%) \| 371 (53.7%) \|  \| \| **Charlson** ≥**3**  N (%) \|  \|  \|  \|  \|  \| **<0.001** \| \| yes \| 70 (40.7%) \| 82 (48.0%) \| 76 (43.9%) \| 113 (65.7%) \| 341 (49.6%) \|  \| \| no \| 102 (59.3%) \| 89 (52.0%) \| 97 (56.1%) \| 59 (34.3%) \| 347 (50.4%) \|  \| \| **Gender**  N (%) \|  \|  \|  \|  \|  \| **0.001** \| \| Male \| 119 (69.2%) \| 102 (59.0%) \| 87 (50.3%) \| 87 (50.3%) \| 395 (57.2%) \|  \| \| Female \| 53 (30.8%) \| 71 (41.0%) \| 86 (49.7%) \| 86 (49.7%) \| 296 (42.8%) \|  \| \| **Immunodepression**  N (%) \|  \|  \|  \|  \|  \| **0.012** \| \| yes \| 9 (5.2%) \| 8 (4.6%) \| 11 (6.4%) \| 23 (13.3%) \| 51 (7.4%) \|  \| \| no \| 163 (94.8%) \| 165 (95.4%) \| 162 (93.6%) \| 150 (86.7%) \| 640 (92.6%) \|  \| \| Initial assessment \| \| \| \| \| \| \| \| **Confusion**  N (%) \|  \|  \|  \|  \|  \| **0.001** \| \| yes \| 7 (4.1%) \| 5 (2.9%) \| 3 (1.7%) \| 18 (10.5%) \| 33 (4.8%) \|  \| \| no \| 165 (95.9%) \| 168 (97.1%) \| 169 (98.3%) \| 153 (89.5%) \| 655 (95.2%) \|  \| \| **eGFR < 60 ml/min/m2**  N (%) \|  \|  \|  \|  \|  \| **<0.001** \| \| yes \| 24 (14.0%) \| 29 (16.9%) \| 42 (24.3%) \| 66 (38.4%) \| 161 (23.4%) \|  \| \| no \| 148 (86.0%) \| 143 (83.1%) \| 131 (75.7%) \| 106 (61.6%) \| 528 (76.6%) \|  \| \| **Oximetry at room air < 94%**  N (%) \|  \|  \|  \|  \|  \| 0.257 \| \| yes \| 47 (28.1%) \| 62 (38.3%) \| 52 (31.1%) \| 50 (31.6%) \| 211 (32.3%) \|  \| \| no \| 120 (71.9%) \| 100 (61.7%) \| 115 (68.9%) \| 108 (68.4%) \| 443 (67.7%) \|  \| \| **SBP (mmHg)** \|  \|  \|  \|  \|  \| 0.845 \| \| Mean (SD) \| 133 (20) \| 132 (23) \| 134 (25) \| 134 (26) \| 133 (24) \|  \| \| Median (Q1, Q3) \| 133.0 (120.0, 146.0) \| 131.0 (116.0, 145.0) \| 132.0 (116.0, 150.0) \| 134.0 (119.0, 148.0) \| 132.0 (118.0, 147.0) \|  \| \| N (% Missing) \| 169 (1.7%) \| 168 (2.9%) \| 171 (1.2%) \| 169 (2.3%) \| 677 (2.0%) \|  \| \| **DBP (mmHg)** \|  \|  \|  \|  \|  \| 0.639 \| \| Mean (SD) \| 81 (13) \| 80 (15) \| 80 (15) \| 79 (16) \| 80 (15) \|  \| \| Median (Q1, Q3) \| 82.0 (72.0, 90.0) \| 81.0 (70.5, 89.0) \| 79.0 (69.0, 91.0) \| 79.0 (67.0, 90.0) \| 80.0 (70.0, 90.0) \|  \| \| N (% Missing) \| 169 (1.7%) \| 168 (2.9%) \| 171 (1.2%) \| 169 (2.3%) \| 677 (2.0%) \|  \| \| **Hear Rate (bpm)** \|  \|  \|  \|  \|  \| 0.952 \| \| Mean (SD) \| 93 (17) \| 93 (16) \| 93 (18) \| 94 (18) \| 93 (17) \|  \| \| Median (Q1, Q3) \| 92.0 (80.0, 102.0) \| 93.0 (82.0, 101.0) \| 94.0 (81.0, 105.0) \| 93.0 (81.0, 106.0) \| 93.0 (81.0, 104.0) \|  \| \| N (% Missing) \| 170 (1.2%) \| 170 (1.7%) \| 170 (1.7%) \| 166 (4.0%) \| 676 (2.2%) \|  \| \| **Heart rate >100 beats/min**  N (%) \|  \|  \|  \|  \|  \| 0.551 \| \| yes \| 57 (33.5%) \| 53 (31.2%) \| 65 (38.2%) \| 60 (36.1%) \| 235 (34.8%) \|  \| \| no \| 113 (66.5%) \| 117 (68.8%) \| 105 (61.8%) \| 106 (63.9%) \| 441 (65.2%) \|  \| \| **Brain natriuretic peptide > 125 pg/ml**  N (%) \|  \|  \|  \|  \|  \| **<0.001** \| \| yes \| 62 (38.0%) \| 67 (41.6%) \| 75 (46.3%) \| 99 (64.3%) \| 303 (47.3%) \|  \| \| no \| 101 (62.0%) \| 94 (58.4%) \| 87 (53.7%) \| 55 (35.7%) \| 337 (52.7%) \|  \| \| **Troponine T > 14 ng/L**  N (%) \|  \|  \|  \|  \|  \| **<0.001** \| \| yes \| 42 (25.9%) \| 43 (26.4%) \| 53 (32.7%) \| 78 (50.0%) \| 216 (33.6%) \|  \| \| no \| 120 (74.1%) \| 120 (73.6%) \| 109 (67.3%) \| 78 (50.0%) \| 427 (66.4%) \|  \| \| **Lactate dehydrogenase > 250 U/L**  N (%) \|  \|  \|  \|  \|  \| 0.417 \| \| yes \| 115 (69.3%) \| 103 (61.7%) \| 111 (68.1%) \| 109 (69.0%) \| 438 (67.0%) \|  \| \| no \| 51 (30.7%) \| 64 (38.3%) \| 52 (31.9%) \| 49 (31.0%) \| 216 (33.0%) \|  \| \| **C-reactive protein > 10 mg/dL**  N (%) \|  \|  \|  \|  \|  \| 0.846 \| \| yes \| 48 (27.9%) \| 48 (27.7%) \| 53 (30.6%) \| 54 (31.2%) \| 203 (29.4%) \|  \| \| no \| 124 (72.1%) \| 125 (72.3%) \| 120 (69.4%) \| 119 (68.8%) \| 488 (70.6%) \|  \| \| **Procalcitonin > 0.5 ng/mL**  N (%) \|  \|  \|  \|  \|  \| **0.001** \| \| yes \| 5 (3.0%) \| 10 (6.0%) \| 14 (8.5%) \| 23 (14.7%) \| 52 (8.0%) \|  \| \| no \| 160 (97.0%) \| 156 (94.0%) \| 151 (91.5%) \| 133 (85.3%) \| 600 (92.0%) \|  \| \| **Ferritin > 500 mg/L**  N (%) \|  \|  \|  \|  \|  \| **0.025** \| \| yes \| 112 (66.7%) \| 102 (61.4%) \| 106 (62.4%) \| 86 (50.9%) \| 406 (60.3%) \|  \| \| no \| 56 (33.3%) \| 64 (38.6%) \| 64 (37.6%) \| 83 (49.1%) \| 267 (39.7%) \|  \| \| IL6 > 10 pg/ml  N (%) \|  \|  \|  \|  \|  \| 0.424 \| \| yes \| 94 (72.3%) \| 99 (75.6%) \| 92 (73.0%) \| 94 (72.9%) \| 379 (73.4%) \|  \| \| no \| 36 (27.7%) \| 32 (24.4%) \| 34 (27.0%) \| 35 (27.1%) \| 137 (26.6%) \|  \| \| **Lymphopenia (<1000/mm^3^)** \|  \|  \|  \|  \|  \| 0.817 \| \| yes \| 86 (50.0%) \| 79 (45.7%) \| 81 (46.8%) \| 86 (49.7%) \| 332 (48.0%) \|  \| \| no \| 86 (50.0%) \| 94 (54.3%) \| 92 (53.2%) \| 87 (50.3%) \| 359 (52.0%) \|  \| \| **Clinical Outcomes** \| \| \| \| \| \| \| \| **ICU admission**  N (%) \|  \|  \|  \|  \|  \| 0.521 \| \| yes \| 22 (12.8%) \| 23 (13.3%) \| 19 (11.0%) \| 15 (8.7%) \| 79 (11.4%) \|  \| \| no \| 150 (87.2%) \| 150 (86.7%) \| 154 (89.0%) \| 158 (91.3%) \| 612 (88.6%) \|  \| \| **Invasive mechanical ventilation**  N (%) \|  \|  \|  \|  \|  \| 0.770 \| \| yes \| 15 (8.7%) \| 13 (7.5%) \| 14 (8.1%) \| 10 (5.8%) \| 52 (7.5%) \|  \| \| no \| 157 (91.3%) \| 160 (92.5%) \| 159 (91.9%) \| 161 (94.2%) \| 637 (92.5%) \|  \| \| **Death** \|  \|  \|  \|  \|  \| **0.003** \| \| yes \| 9 (5.2%) \| 10 (5.8%) \| 7 (4.0%) \| 24 (13.9%) \| 50 (7.2%) \|  \| \| no \| 163 (94.8%) \| 163 (94.2%) \| 166 (96.0%) \| 149 (86.1%) \| 641 (92.8%) \|  \| |

SBP: systolic blood pressure; DBP: dyastolic blood pressure; eGFR: estimated glomerular filtration rate (by CKD-EPI formula); ICU: intensive care unit

**Suppl. Table 3. Baseline characteristics across the deaths status.**

|  | Survivors  [n=641] | Deceased  [n=50] | p-value* |
| --- | --- | --- | --- |
| **Demographics** | | |  |
| Age (years), median (IQR) | 61 (48-74) | 80 (72-86) | <0.001 |
| Age > 65, % | 274/641 (42.7) | 46/50 (92.0) | <0.001 |
| Males, % | 361/641 (56.3) | 34/50 (68.0) | 0.137 |
| Vaccine status^a^  Complete  Partial | 34/641 (5.3%)  12/641 (1.9%) | 1/50 (2.0%)  1/50 (2.0%) | 0.590 |
| Comorbidities | | |  |
| Diabetes, % | 132/641 (20.6) | 23/50 (46.0) | <0.001 |
| Hypertension, % | 291/641 (45.4) | 37/50 (74.0) | <0.001 |
| Chronic respiratory disease | 109/640 (17.0) | 14/50 (28.0) | 0.051 |
| Smoker (current or former), % | 50/498 (10.0) | 2/40 (5.0%) | 0.558 |
| Charlson comorbidity index ≥3, % | 296/638 (53.6) | 45/50 (90.0) | <0.001 |
| Obesity (BMI ≥30), % | 178/446 (39.9) | 12/33 (36.4) | 0.688 |
| Initial assessment | | |  |
| Oximetry at room air < 94%, % | 185/609 (30.4) | 26/45 (57.8) | <0.001 |
| Lymphopenia (<1000/mm^3^), % | 296/641 (46.2) | 36/50 (72.0) | <0.001 |
| Troponine T > 14 ng/L | 181/598 (30.3) | 35/45 (77.8) | <0.001 |
| Brain natriuretic peptide > 125 pg/ml, % | 264/595 (44.4) | 39/45 (86.7) | <0.001 |
| Clinical presentation | | |  |
| Days of symptoms before admission, median (IQR) | 7.0 (4-10) | 6.0 (3-8) | 0.380 |
| Dyspnea, % | 374/639 (58.5) | 32/50 (64.0) | 0.449 |
| Radiological characteristics  Bilateral pneumonia, %  Unilateral pneumonia, % | 226/634 (35.6)  318/634 (50.2) | 10/49 (20.4)  32/49 (65.3) | 0.078 |
| Opacities >50% of lung surface on X-Rays,% | 143/641 (22.3) | 12/50 (24.0) | 0.782 |
| Clinical Outcomes | | |  |
| Length hospital stay (days), median (IQR) | 8 (5-12) | 11.5 (6-25) | 0.002 |
| Non-invasive respiratory support, % | 140/641 (22.1) | 32/50 (64.0) | <0.001 |
| ICU admission, % | 63/641 (9.8) | 16/50 (32.0) | <0.001 |
| Length ICU stay (days), median (IQR) | 9.0 (5-15) | 18 (8-36) | 0.062 |
| Invasive mechanical ventilation, % | 37/641 (5.8) | 15/50 (30.0) | <0.001 |

ICU: intensive care unit; IMV: invasive mechanical ventilation; IQR: interquartile rate; *, U Mann-Whitney and Chi Square test, as appropriate.

^a^ We defined complete vaccination (CV) as symptom onset after 14 days of the second dose of vaccines (a single Janssen dose) and partial vaccination (PV) as administration of only the first dose, or symptom onset within 13 days after the second dose (single dose in Janssen).
