# Supplementary material for: Early carbohydrate antigen 125 as a mortality predictor in hospitalized patients with coronavirus disease 2019
Source: Front Cardiovasc Med. 2022 Oct 20;9:941512. doi: 10.3389/fcvm.2022.941512 (PMC9631479; doi:10.3389/fcvm.2022.941512)
Supplement: Supplementary file 2 [file Data_Sheet_2.PDF]

COVID19-**ALC** research group

Early CA125 as a mortality predictor in hospitalized patients with COVID-19.

A case series of 691 Mediterranean patients.

POPULATION, SETTINGS

691

Tertiary referral hospital

Age 63 years
Males 57.2%
Charlson comorbidity index ≥3 46.6%
Hypertension 47.5%
Obesity 39.7%
Diabetes 22.4%

Pneumonia 85.8%
Hypoxemia 32.3%

EXPOSURE

Stored biobank samples

CA125 determination (Elecsys CA125 II assay-[Roche Diagnostics GmbH])

Wide availability

Low Cost

easy widespread implementation in clinical practice

PRIMARY OUTCOMES

In-hospital Mortality
Invasive Mechanical Ventilation
Non-invasive respiratory support

CA125 biomarker

Congestion
Inflammation

FINDINGS

Mortality

Risk Factor	Odds Ratio (95% CI)	p
≥65 years of age	**31.03**	0.004
Diabetes	**2.58**	0.026
Immunosuppression	**3.96**	0.024
CA125 > 15.5 U/mL (75th percentile)	**2.83**	0.017

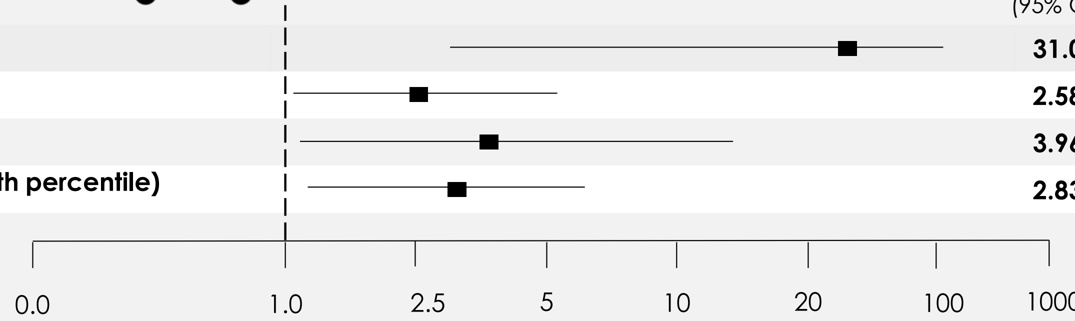

Independent associated covariates in multiple logistic regression model

Gradient of risk of CA125 for predicting

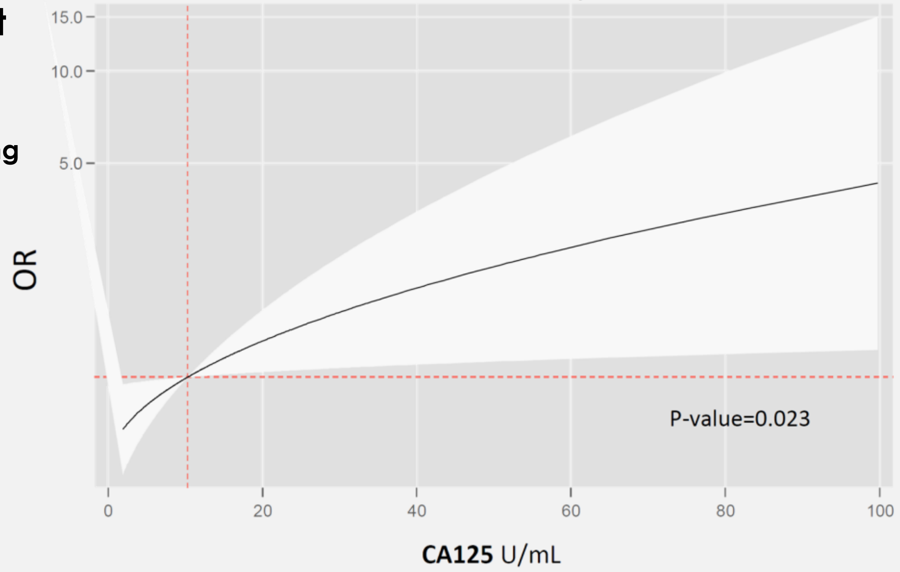

Multivariate analysis by fractional polynomials

CA125

X

Invasive Mechanical Ventilation

Non-invasive respiratory support

Moreno-Perez O. et al.
